# Evaluation of self‐regulation by the hunting community: A case study on the voluntary restraint of woodcock hunting in the UK

**DOI:** 10.1002/ece3.11377

**Published:** 2024-05-16

**Authors:** Cat M. McNicol, Matt B. Ellis, Joah R. Madden

**Affiliations:** ^1^ British Association for Shooting & Conservation Marford Mill Rossett UK; ^2^ University of Exeter Exeter UK

**Keywords:** behaviour change, compliance, hunting, messy data, woodcock

## Abstract

Behaviour change through voluntary action can be an important approach to reducing human impacts on biodiversity. One example is self‐regulation in hunting, potentially a vital contributory factor in improving the sustainability of wild bird harvest. There has been a growing realisation among woodcock *Scolopax rusticola* hunters, reinforced by advice from sector organisations, that components of the UK woodcock populations are declining and that some aspects of woodcock hunting, specifically timing of harvest, may contribute to these. This study utilised five qualitative and quantitative data sets, collected for different purposes, to assess the behaviour and attitudes of woodcock hunters, both currently and over the past century. In the UK, relatively few woodcock are harvested and few hunters or species‐specific shoots target them. An estimated 26%–29% of lowland shoots advertise or harvest woodcock, with fewer than 5% of shoots or hunters participating in ‘woodcock specific’ shoot days. The number of birds harvested has fallen in recent years and is estimated to be between 62,000 and 140,000. Qualitative data suggests that over 90% of hunters now report shooting woodcock only after the recommended date of 1st December, or not at all. This is reflected in bag data which shows that, since 2018, fewer than 3%–13% of woodcock shot were harvested prior to 1st December. Around a third of hunters have reported stopping shooting woodcock and it is likely the harvest will decline in coming years due to voluntary restraint. This work demonstrates both through self‐report data and independent harvest data that behaviour change among hunters can be effected. This provides a working example where self‐regulation in response to a collective sector‐led effort has the potential to conserve wild quarry.

## Graphical abstract

A range of ‘messy data’ indicates that UK hunters have attended to calls for voluntary self‐regulation when hunting woodcock. In the past couple of decades, the overall number of woodcock shot has declined, the proportion of hunters is decreasing and, critically, the proportion of woodcock shot early in the season, before December 1, when resident breeding birds are more likely to be shot, has declined. This indicates that behaviour change of the hunting community can be effected by clear messaging and voluntary restraint.

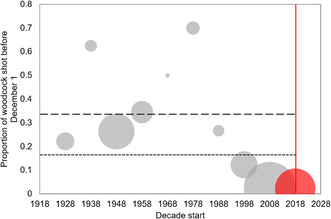

## 1 INTRODUCTION

There is an increasing appreciation of bottom‐up, community‐led change in conservation, in contrast to traditional top‐down regulation. This change acts primarily on a discrete level, whereby understanding and decision‐making by individual people can collectively drive action (Barr et al., 2011). Achieving the intended outcomes must be driven not only by education and awareness raising, but a subsequent and appropriate level of behaviour change by engaged parties as a product/consequence of voluntary compliance and self‐regulation (Barr et al., 2011; McDonald et al., 2014; Reddy et al., 2017). As behaviour change manifests on an individual level, it can gather momentum and force change at group and societal levels, ultimately resulting in shifts in societal perception and policy (Whitmarsh et al., 2021). Approaches to such campaigns may differ subject to the target audience and the rate of change required (Barr et al., 2011). Voluntary compliance and self‐regulation have been frequently proposed to counter the current threats of biodiversity loss and environmental degradation (Barr et al., 2011; Reddy et al., 2017; Travers et al., 2021; Whitmarsh et al., 2021). However, the evaluation of conservation interventions or campaigns aimed at changing behaviour is rarely undertaken due to limited appetite or funding (Ferraro & Pattanayak, 2006; Travers et al., 2021). Yet, this is a vital step in understanding how successful messaging has been and, when motivations are well understood, how sustainable any behaviour change is likely to be (Reddy et al., 2017; Steg & Vlek, 2009).

Appraisal of conservation messaging and associated behaviour change requires a mixed methods approach which incorporates both qualitative and quantitative data (Travers et al., 2021). In many instances, conservation messaging is not undertaken with specific metrics for measuring success in place, making such evaluation challenging. As a result, in more recent years, conservationists have begun utilising data which has been collected for different purposes under informal study designs, that is ‘messy data’ (Dobson et al., 2020). Use of multiple ‘messy’ data sources can produce a high volume of low‐cost data to answer novel research questions through ‘data triangulation’, that is, combining multiple methods or sources of data about the same subject (Carter et al., 2014; Dobson et al., 2020). Each data source may have its inherent strengths and weaknesses; however collectively, data sources can provide a strong descriptive overview of patterns, regardless of background noise present in individual studies (Dobson et al., 2020).

In the light of an increasing focus on the ecological effects of game shooting in the UK (Madden et al., 2023; Madden & Sage, 2020; Mason et al., 2020; Sage et al., 2020), the actions of people who shoot game in the United Kingdom (hereafter ‘hunters’) are being scrutinised and attempts are being made, through both legislation and advice, to change elements of their behaviour to ensure no net ecological loss from their activities. In the case of wild bird harvest (as opposed to game that is reared and released, where the population size and conservation status of released species is dependent on artificial rearing levels), the current approach via legislation to secure positive species conservation statuses is to (i) limit the take of certain species at protected sites through consenting processes or (ii) add or remove species from Schedule II of the Wildlife and Countryside Act (Wildlife and Countryside Act 1981, 2023) which allows harvest during a defined open season. In both instances, these strategies are top‐down, enforcement‐based approaches to changing behaviour. This approach may produce a more immediate and complete change, because the law is clearly defined and non‐compliance is illegal. However, regulation of hunting through legislation may be resisted or defied, perhaps covertly, and so lose efficacy (Schroeder et al., 2021; Watkins et al., 2018). For example, legislation to restrict the use of lead shot for some specific hunting scenarios in the UK was introduced from 1999 in England, but surveys indicate that compliance is currently low (Green et al., 2023; Stroud et al., 2021) contrasting with higher levels of compliance to similar regulations in some parts of the USA and EU (Kanstrup & Balsby, 2019; Mateo & Kanstrup, 2019). An alternative to a legislated enforcement approach is sector‐led, voluntary change which is self‐regulatory and which depends on the buy‐in of stakeholders to deliver benefits through self‐governance. Such self‐regulation has been demonstrated within the recreational fishing sector (Cooke et al., 2013) and among (at least some) hunters outside the UK (Greeley, 2023; Schou & Bregnballe, 2007). Ultimately, this has enabled stakeholders and regulators to work collectively towards common management objectives.

One key scenario in the UK hunting sector where change in hunter behaviour has biodiversity and conservation implications is the harvest of wild Eurasian woodcock *Scolopax rusticola* (hereafter Woodcock). Woodcock are medium‐sized wading birds, adapted to woodland habitats, and present across temperate and subarctic Eurasia (BirdLife International, 2019). This species is largely migratory with breeding occurring in northern latitudes (mainly in Scandinavia, Finland, the Baltic countries and Russia), and with wintering populations occurring widely across temperate Western Europe and the Mediterranean. Within Europe there is also a non‐migratory component, including a resident population in the UK, which from November onwards intermixes with the migratory population with which it is morphologically identical (Hoodless & Coulson, 1994; Powell, 2013). The UK wintering population has remained stable (Frost et al., 2019), but the resident breeding population has declined in distribution by 29% since 1988 (Balmer et al., 2013). The global population is stable and extremely large, with an estimated 10,000,000–26,000,000 individuals dispersed across two continents in the breeding season and three in the winter, and in consequence is listed as Least Concern on the International Union for the Conservation of Nature's Red List at an international and European level (European Commission, 2021; IUCN, 2012).

Woodcock are huntable across the majority of their wintering range with an estimated annual harvest within Europe of 3–4 million birds (Powolny & Czajkowski, 2022). The hunting season for woodcock in the UK runs from 1st October to 31st January (1st September to 31st January in Scotland). There are no harvest limits or restrictions, and the annual harvest was estimated at 140,000 individuals (CI: 120,000–170,000), in 2016, down from 180,000 (CI: 150,000–220,000) in 2004 (Aebischer, 2019). A recent assessment of the sustainability of waterbird hunting in the UK found that the current harvest of the resident and migratory woodcock populations was likely to be sustainable, with only a 10% and 5% probability respectively that current harvest rates could exceed that required to maintain stationary growth although the population estimates on which these calculations were based are deemed unreliable (Ellis & Cameron, 2022).

Currently, there are calls to reduce the open season for woodcock (parallelparliament.co.uk, 2023; UK Government and Parliament, 2023), as well as calls for self‐regulation by hunters following advice from the Game and Wildlife Conservation Trust (GWCT), an organisation that researches game birds in the UK and carries credence for this work with hunters. The advice from GWCT regarding shooting woodcock has developed over the past decade. In early 2015, their position was that shooting was unlikely to be a main factor in declines in UK woodcock populations (https://www.gwct.org.uk/media/672326/Woodcock‐fact‐sheet.pdf). By December 2015, they were advising that hunters should stop shooting woodcock if temperatures fell below freezing for 7 days or if snow is lying and that shoots should exhibit caution in their shooting if woodcock were known to breed locally and that shoots should improve their local knowledge of woodcock populations through spring roding counts (https://www.gwct.org.uk/news/news/2015/december/20151202/). In December 2017, the GWCT advised that due to a poor breeding season, shoots and hunters should ‘rethink their woodcock shooting for this season and reduce their bags’ (https://www.gwct.org.uk/news/news/2017/december/new‐advice‐on‐woodcock‐shooting‐from‐gwct‐expert/). In March 2018, the cold weather advice was updated to cease woodcock hunting after 4 days of sub‐zero temperatures (https://www.gwct.org.uk/news/news/2018/march/shooters‐urged‐to‐take‐%E2%80%9Ccautious‐approach%E2%80%9D‐when‐shooting‐woodcock/). In November 2018, the GWCT advised hunters to ‘refrain from shooting woodcock before December 1’ (https://www.gwct.org.uk/news/news/2018/november/gwct‐woodcock‐expert‐reiterates‐shooting‐advice/). Their current advice is that hunters should avoid shooting woodcock early in the season, until after the migrant birds have arrived, with the 1st December being a useful rule of thumb (Brewin et al., 2022). This call for early‐season restraint was accompanied by advice to better understand local populations, shoot flight lines with caution and limit hunting in severe weather. Ultimately, this recommendation was made to reduce the potential impact of shooting on resident breeding birds, by confining hunting to periods of the season when the number of over‐wintering migrant birds from Eurasia is at their highest (Hoodless & Coulson, 1994; Powell, 2013).

In this study we assess the behaviour of UK woodcock hunters over time and investigate the impact of sector‐led calls for voluntary restraint on the shooting of woodcock. Through utilising multiple data sources, we combine qualitative and quantitative datasets to understand if the shooting sector demonstrates self‐regulation of hunting behaviour. This requires that we understand: (a) the current scale and extent of woodcock shooting in the UK, both to provide a baseline for any future work and to set our findings about harvest behaviour in context of national and international populations and prospects; (b) the current expression of self‐regulation practised when shooting woodcock by UK hunters, suggested by the patterns of seasonality in shooting behaviour or confirmed by survey data, to provide evidence from multiple sources for hunter engagement with advice and to provide a baseline for any future behaviour change; (c) the change in the harvesting behaviour of woodcock hunters over time, from the 1930s through the period when advice on woodcock hunting was issued by the GWCT to the present, to provide evidence of the efficacy of developing self‐regulation and non‐legislative advice. Each of our data sources has limitations and provides partial evidence, but by asking these three questions of multiple datasets, we may improve our confidence in the answers.

## 2 METHODS

We took a triangulation approach to account for the poor data quality available from any single set of sources (Dobson et al., 2020). We deployed five complementary methods, each offering a different perspective on woodcock harvest from hunters and shoots. Each of these datasets provided partial answers to, and corroboration for, our three central questions about: (a) current scale and extent of woodcock shooting in the UK; (b) current seasonality of woodcock harvest in the UK; (c) change in patterns of woodcock harvest over time.

### 2.1 Records from game shoots and hunters

Game shoots and individual hunters commonly record the number of birds of each species that are harvested each day, alongside date, location and often other data such as number of hunters involved or shots fired. This provides a contemporaneous record of harvest, and being typically records for personal memory and reflection have little reason to be dishonest. These game records are usually held privately in personal logs, although some may be published for various reasons. We collected a set of these records via: (a) examination of published literature such as the shooting press describing commercial shooting days or a book that collates gamecards for historical reasons (Percy, 1986); (b) requests to hunters/shoots circulated via the British Association for Shooting and Conservation (BASC) members' magazine, social media and personal contacts. BASC is a membership organisation that represents the interests of shooting sports in the UK and currently comprises ~150,000 individuals. However, the use of social media and personal contacts means that our data sample was not stratified and its representation of UK Guns in general is unclear.

Access to private information was approved by the Exeter University Department of Psychology ethics committee (3041532). All contributors were provided with a briefing sheet which included acknowledgement of consent. Contributor's identities were not transcribed but instead an alpha‐numeric code was used to separate individual contributions. Shoot locations were transcribed at a county level to preserve anonymity.

Records were collected opportunistically between 27 July and 24 September 2023, and we considered data from 1933 to 2022 from shoots within the UK and pooled data by decade, starting on the eighth year of each, for example 1948, 2008, so that the final decade ran 2018 (when the GWCT advice, on refraining from shooting before December 1, was issued) to the end of the 2022/23 shooting season (please see accompanying dataset for breakdown of records/decade). We excluded shoots that were predominantly upland (which we deemed to be indicated by >1/3 of the harvest being red grouse *Lagopus lagopus*). We excluded shoots that were predominantly wildfowling (which we deemed to be indicated by >1/2 the harvest being ducks/geese). These were excluded because the methods of shooting (e.g. over decoys or flight ponds) and/or habitats (moorland or coast) were hard to compare with the majority of the more common lowland for which we had records. We also excluded records that were illegible, or which did not contain all elements of the key information (bag number per species, date at the precision of month and year).

We included 1750 records of lowland shooting days in the UK. These included records from 1003 days from 258 different shoots that recorded the collective effort on the shoot day (all birds harvested) from shoots where >20 birds were shot. Records from these shoots were not evenly distributed across time (Decade starting 1928:61 records; 1938:117; 1948:117; 1958:112; 1968:20; 1978:41; 1988:45; 1998:89; 2008:222; 2018:179). These comprised 725 records from days of 20–113 birds (small shoots = 72% vs. 71% nationally (following classifications described in Madden, 2021)); 72 from days of 114–190 birds (medium shoots = 7% vs. 18% nationally); 2064 from days of greater than 190 birds (large shoots = 21% vs. 10% nationally). These are an overrepresentation of large shoots and underrepresentation of medium shoots considering their prevalence in the UK.

The remaining 747 records were of days where either the personal rather than collective bag was reported, or where fewer than 20 birds were reported harvested. These included 85 shoots. The size of these shoots could not be assessed due to lack of total bag size.

This data set allowed us to consider the current scale and extent of woodcock shooting in the UK and change in patterns of woodcock harvest over time.

### 2.2 Wing survey

Since the 2017/2018 hunting season, hunters have been encouraged to voluntarily submit woodcock wings to the BASC wing survey (BASC, 2023). One wing was removed from each harvested bird as close to the body as possible, including all of the tertial and axillary feathers. The age of each bird (adult/juvenile) was assessed by trained staff based on plumage characteristics (Baker, 2016). Wings which had been submitted with the location and exact date or month the bird was harvested were included in the assessment.

A total of 537 woodcock wings were submitted between 2017 and 2023, each representing an individual bird. Approximately half (*n* = 291) of the wings submitted specified the exact date and country where the bird was harvested and could be used to assess compliance with the voluntarily shortened season. The remaining samples had no accompanying date (*n* = 244) or had no location associated with them (*n* = 2) and could not be used in this assessment. Wings were submitted from Scotland, England, Wales and Northern Ireland, suggesting broad representation of the hunting community across the UK. The number of birds harvested in each month was summed across the 6 years to give an average percentage of woodcock harvested in (i) each month of the open season and (ii) in each UK country across these years.

This data set allowed us to consider current seasonality of woodcock harvest in the UK.

### 2.3 Records from advertised game shoots

We searched the online adverts posted by game shoots on the website GunsOnPegs (www.gunsonpegs.com) on 9th October 2023. The website hosts advertisements from around 1000 game shoots, sporting agents and syndicates in the UK (an estimated 10% of the UK total).

The website database was searched under the ‘Find Shooting’ option. The quarry filter was set to ‘pheasant’ to obtain an estimate of the total number of lowland game shoots within each region of the UK, excluding entries from sporting agents, who typically cover a number of different shoots. The quarry filter was then set to ‘woodcock’ to obtain an estimate of the number of shoots offering woodcock as one of their quarry (again, excluding entries from sporting agents).

The written descriptions of all shoots advertising woodcock as a quarry were then searched. Those which: (a) listed woodcock as their primary or secondary quarry OR; (b) stated that they offered driven woodcock shooting OR; (c) had the word woodcock in their title were counted as ‘woodcock specific’ shoots. Results were transcribed with anonymised shoot names and the location was recorded at the region level.

This dataset allowed us to consider the current scale and extent of woodcock shooting in the UK.

### 2.4 GunsOnPegs shoot census

Each year since 2013 GunsOnPegs has run an online survey of shoot providers and hunters, named the ‘Shoot Census’. There are a core set of questions in this survey aimed at understanding buying intentions and brand awareness as well as the health, economics and activity of the gamebird shooting sector. The survey is promoted by a consortium of partner groups (including BASC, GunsOnPegs and other major shooting organisations) via social media and email. Participation is usually incentivised with entry into a draw for shooting equipment. Typically, the survey is answered by over 500 shoots and 5000 participants responding to requests from partner organisations. Each year, questions are asked about the provision and experience of shooting, but the survey authors may also supplement the standard questions with questions on topical subjects. In 2016 and 2018, these included questions about woodcock shooting.

In 2016, the participant survey included additional questions, specifically asking ‘Do you shoot woodcock?’ (‘Yes – I do shoot them, when asked, but don't really understand the conservation issues’, ‘Yes – I do shoot them but only when it is clear there is a clear understanding of the local population’, ‘No – In the last 3 years I decided to stop shooting; even when asked/available’, ‘No – I stopped shooting them more than three years ago; even when asked/available’, ‘I have never shot woodcock’), ‘What is your favourite quarry to shoot?’ and ‘Please select the three quarry you shoot most often’. In 2018 the shoot provider survey asked ‘Which of the following species and game types are present on the shoot?’ and the participants were asked ‘Which species did you shoot last season?’. The raw data for each shoot census is made available to participating partner organisations, and so we (CMM & MBE at BASC) extracted the total number of respondents in 2016 and 2018, as well as their responses to the above questions.

This dataset allowed us to consider the current scale and extent of woodcock shooting in the UK and indications of current self‐regulation of woodcock harvest in the UK.

### 2.5 Value of shooting

Between 29th July and 11th October 2022, a sector‐wide survey was sent to all members of all organisations representing live‐quarry hunters and target shooters in the UK. This survey had a ‘quick survey’ followed by a set of detailed questions. Within the second part of the survey, individual respondents were asked to report their harvest of woodcock in the 2021/22 season. In addition, hunters were asked when they shoot woodcock (‘I shoot woodcock from 1st October’, ‘I shoot woodcock from 1st December’, ‘I do not shoot woodcock because I am following voluntary restraint’, ‘I do not have access to woodcock shooting’). Providers of shooting opportunities were also asked whether they provide access to woodcock shooting (‘I provide woodcock shooting from 1st October’, ‘I provide woodcock shooting from 1st December’, ‘woodcock are present but I have imposed voluntary restraint on the shooting of them’, ‘Woodcock are present and I do not provide woodcock shooting’).

The survey was completed by 11,227 participants, and 2867 answered the questions on attitudes to woodcock shooting. The shooting provider survey was completed by 703 providers, and 106 answered the woodcock question. The estimated national harvest of woodcock was calculated by grossing up the average number of woodcock harvested per participant to the total number of shooting participants in the UK. The upper estimate for the number of participants was taken as the number of people holding a shotgun or firearm certificate, the central estimate was scaled down based on the number of shotgun or firearm certificates who reported being actively involved in live‐quarry shooting (82%) and the lower estimate was set at 18% below the central estimate.

This dataset allowed us to consider the current scale and extent of woodcock shooting in the UK, the current seasonality of woodcock harvest in the UK and the current self‐regulation of woodcock harvest in the UK.

## 3 RESULTS

### 3.1 Game cards

#### 3.1.1 Current scale and extent of woodcock harvest in the UK

In total, the reported bags described the harvest of 116,017 birds including 1015 woodcock (0.87% of birds harvested). Of the 1750 records considered, woodcock were reported harvested in 310 of them (17.4%). The distribution of the number of woodcock harvested was highly skewed. One record comprised 164 woodcock harvested in a single day; the largest reported harvest of woodcock in Wales or England, made in 1963 in Wales. We removed this record from subsequent analyses because it was an extreme outlier. There were 15 records where woodcock comprised >33% of the bag, constituting what might be considered to be ‘woodcock specific’ days (0.86%).

Of the 118 shoots for which we had gamecard records from 2013 to the present, 13 did not include woodcock on the potential quarry list even though they listed other wild game, while a further 15 did not include woodcock but also did not list other wild game. Therefore, we consider that between 11% and 24% of game shoots do not shoot woodcock or if so, do so very intermittently. Some of these shoots where woodcock are not harvested may be where the species is absent.

#### 3.1.2 Current seasonality of woodcock harvest

Of the 183 harvested woodcock included on the gamecards from 2018 and 2023, only 4 (2.2%) were recorded as being harvested before 1st December. These were harvested at 3 of the 32 (9.3%) shoots where any woodcock were recorded as harvested. For all three of these shoots, only a single woodcock was harvested on any 1 day before 1st December.

#### 3.1.3 Changes in patterns of woodcock harvest over time

**FIGURE 1:**
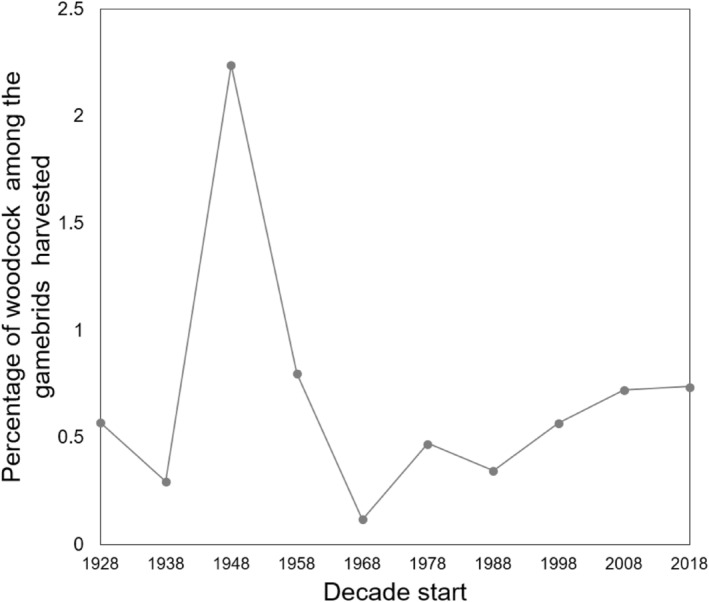
The percentage of woodcock shot as part of the total gamebird harvest, reported in each decade.

There has been little change in the number of shoots where woodcock were reported harvested over the past 90 years, with 26% (65/249) shoots for which we had records from 1930 to 2017 reporting that woodcock were harvested there on any day compared with 30% (30/100/) shoots with records from 2018 to 2023. Equally, there has been little change in the absolute proportion of the total harvest that is woodcock, with them exceeding 1% of the total harvest of gamebirds in only one decade (1948–1958) (Figure 1). The current level of 0.74% (183/24,829) from 2018 to 2023 is almost identical to the overall mean proportion of 0.74% (668/91,024) reported harvested from 1933 to 2017.

**FIGURE 2:**
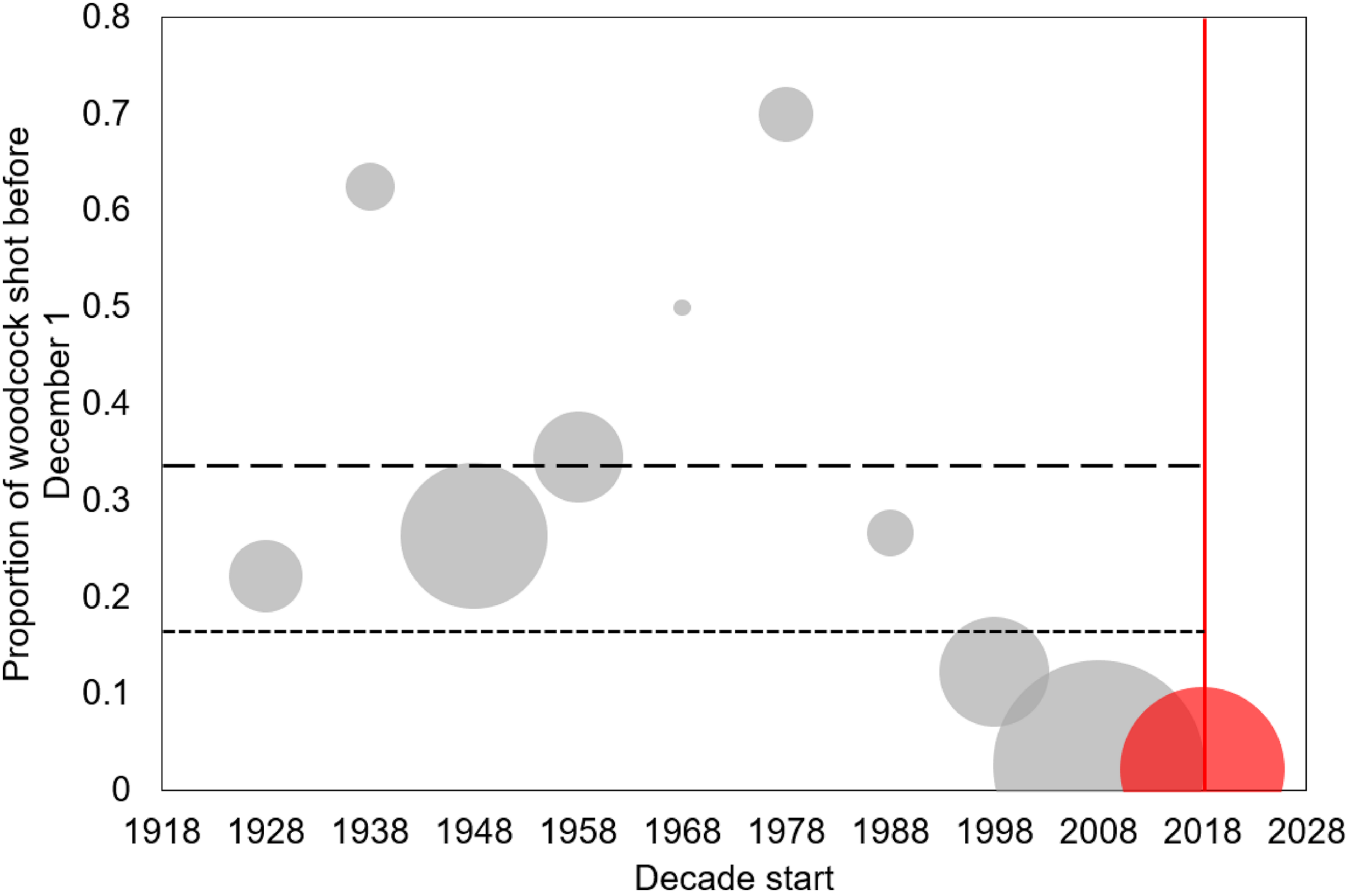
The proportion of woodcock harvested before 1st December in a decade. Point size indicates the total number of woodcock harvested that decade. Long‐dash line shows the unweighted mean pre‐1st December harvest before 2018. Short‐dash line shows the weighted mean pre‐1st December harvest before 2018. Red line demarks when the GWCT advice on not shooting pre‐1st December was published. Red point shows the proportion shot post 2018.

However, the pattern of harvest within years has changed markedly. There has been a general decrease in the proportion of woodcock that are harvested before 1st December over the past 90 years (Figure 2). When we explicitly looked at changes post the specific advice from GWCT in 2018, we found that prior to 2018 16.8% (weighted average – accounting for sample size per decade) – 34% (unweighted average – considering only the raw percentage per decade) of all woodcock harvested in a decade were harvested prior to 1st December, whereas post 2018, only 2.2% were harvested prior to 1st December. However, it is interesting to note that in the preceding decade (2008–2017), during which time GWCT advice was developing but did not specify 1st December as a rule of thumb, only 2.7% of woodcock were harvested prior to 1st December, a four‐fold decrease from the decade preceding that (1998–2007) which itself was half that of the period 1988–1997.

### 3.2 Wing survey

#### 3.2.1 Current seasonality of woodcock harvest

From this sample, there were no woodcock harvested in October, 13% of woodcock (*n* = 38) were harvested in November, with 29% (*n* = 84) harvested in December, and the majority (58%, *n* = 169) harvested in January (Tables 1 and 2).

**TABLE 1:** Percentage of woodcock submitted to the BASC wing survey harvested in each month of the hunting season within each country of the United Kingdom (*n* = 291).

Month	Percentage of birds harvested				Total
	England	N. Ireland	Scotland	Wales	
October	0	0	0	0	0
November	6	34	1	6	13
December	38	37	0	71	29
January	56	29	99	23	58

**TABLE 2:** Number of woodcock submitted to the BASC wing survey harvested each month of the hunting season in the United Kingdom (*n* = 291).

Season	Month				Total
	October	November	December	January	
2017/2018	0	0	0	2	2
2018/2019	0	4	43	84	131
2019/2020	0	22	12	48	82
2020/2021	0	3	11	3	17
2021/2022	0	7	8	28	43
2022/2023	0	2	10	4	16
Total	0	38	84	169	291

### 3.3 Records from advertised game shoots

#### 3.3.1 Current scale and extent of woodcock harvest in the UK

**FIGURE 3:**
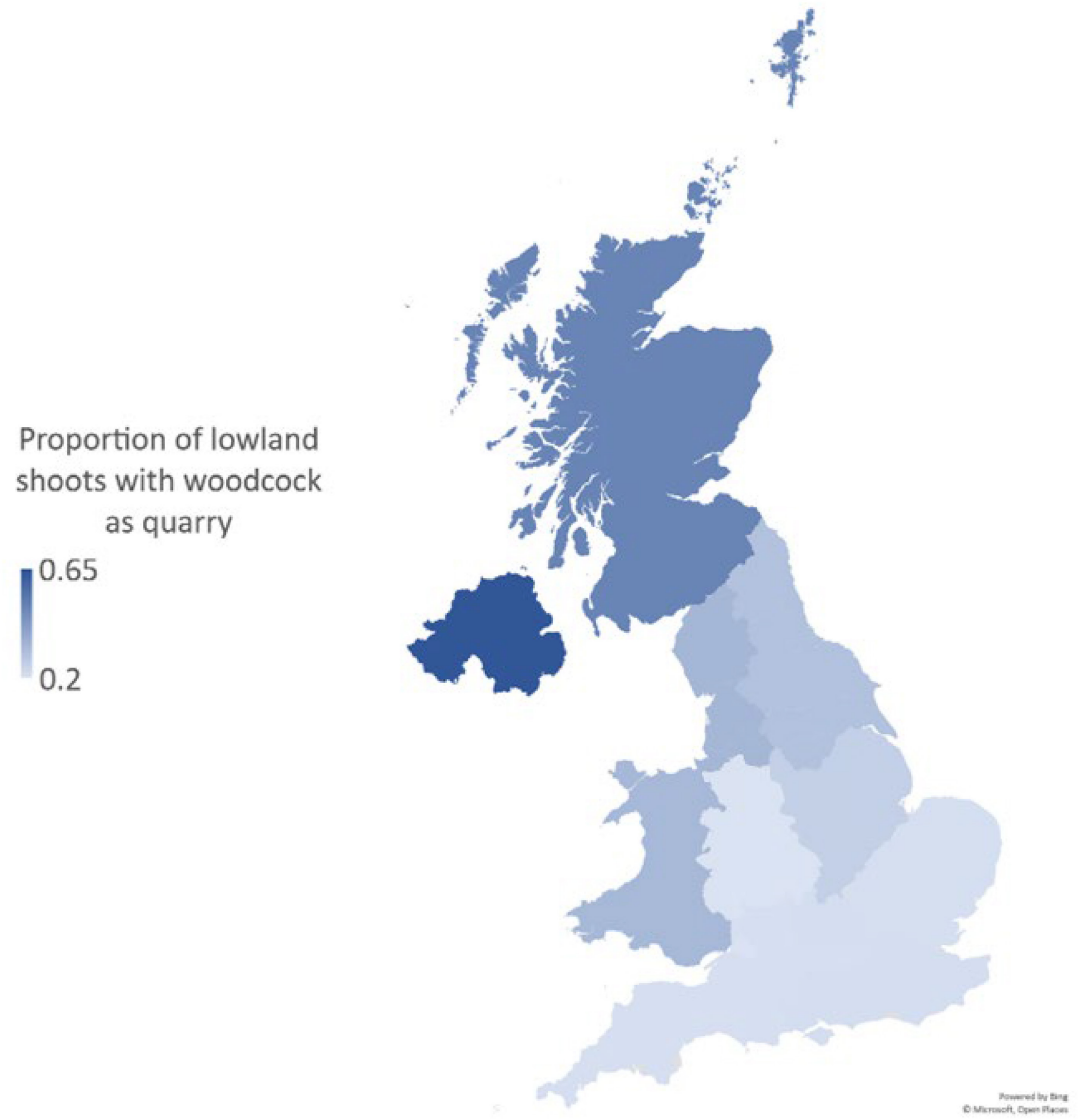
Proportion of lowland game shoots that offer woodcock as quarry, in each region of the United Kingdom as reported on GunsOnPegs.

**FIGURE 4:**
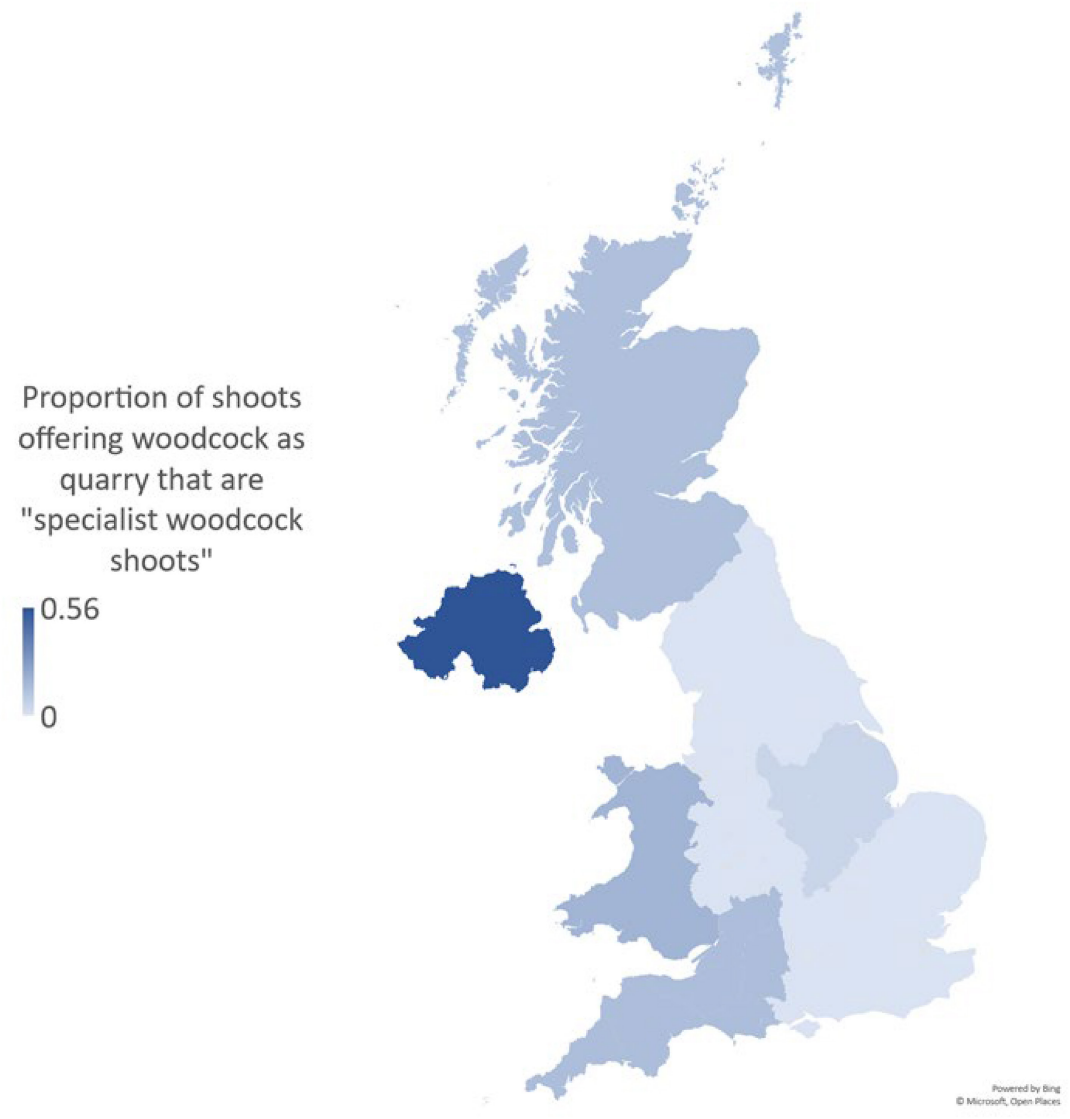
Proportion of lowland game shoots offering woodcock as quarry that include woodcock as a focal quarry species for the day (driven or primary/secondary quarry species), in each region of the United Kingdom as reported on GunsOnPegs.

Of the 959 lowland game shoots advertising on GunsOnPegs, 273 (28.5%) offered woodcock as a quarry species. Proportionately, woodcock as a quarry species was most common in Northern Ireland, Scotland, North‐West England, Wales and North‐East England (Figure 3). Twenty‐six of these advertising shoots offer woodcock as a primary or driven quarry. This constitutes just under 3% of the advertised lowland game shoots, although some of these woodcock specialist shoots are in upland regions of Scotland, Wales or the South‐West of England. These woodcock specialist shoots are concentrated in Northern Ireland, Wales, South‐West England and Scotland (Figure 4).

### 3.4 GunsOnPegs shoot census

#### 3.4.1 Current scale and extent of woodcock harvest in the UK

From the 2016 GunsOnPegs survey, 11.4% of 4492 hunters reported harvesting woodcock as one of their three most common quarry during the preceding year, and 4.3% (of 4205 responding hunters) reported that woodcock were their preferred quarry from a set of 12 choices. From the 2018 GunsOnPegs survey, 32.9% of 9562 responding hunters had harvested woodcock during the previous season and 82.5% of the 652 responding shoot owners indicated they had woodcock present on their land.

#### 3.4.2 Current self‐regulation of woodcock harvest

From the 2016 GunsOnPegs survey, almost two‐thirds of hunters (63.8% of 4113 respondents) reported that they do or would shoot woodcock, with 84.1% of those hunters stating that they shoot them ‘only when it is clear there is good understanding of the local population’. Fifteen percent of hunters have never shot woodcock while 21.3% have ceased shooting woodcock even when asked to/available.

#### 3.4.3 Changes in patterns of woodcock harvest over time

From the 2016 GunsOnPegs survey 11.8% of 4113 respondents reported that they had stopped shooting woodcock in the 3 years preceding the survey.

### 3.5 Value of shooting

#### 3.5.1 Current scale and extent of woodcock harvest in the UK

The overall woodcock harvest in 2019/20 was estimated at 62,000 birds with the majority harvested in England (37,000; Table 3).

**TABLE 3:** Estimated breakdown of the 2019/20 woodcock harvest by country in the UK.

Country	Estimated bag
England	37,000 (31,000–43,000)
Scotland	11,000 (9000–13,000)
Wales	7000 (6000–9000)
Northern Ireland	7000 (5000–8000)
United Kingdom	62,000 (51,000–73,000)

*Note*: Numbers in parentheses represent estimated bags based on lower and upper estimates of the number of shooting participants in the United Kingdom.

#### 3.5.2 Current self‐regulation of woodcock harvest

Attitudes to woodcock shooting were similar for both participants and providers with 72% of participants and 74% of providers either choosing not to shoot woodcock at all, or not having access to woodcock shooting (Table 4). For those who did shoot woodcock at all, compliance with the recommended best‐practice dates for shooting woodcock was also similar for both participants (20%) and providers (19%). Removing those who do not have access to woodcock shooting the levels of non‐compliance were 8% for participants and 7% for providers.

**TABLE 4:** Attitudes of shooting participants (*n* = 2867) and providers of shooting opportunities (*n* = 106) to the shooting of woodcock in the United Kingdom.

Survey answer	Participants (%)	Providers (%)
I shoot woodcock from 1st October I provide woodcock shooting from 1st October	8	7
I only shoot woodcock after 1st December I only provide woodcock shooting after 1st December	20	19
I do not shoot woodcock because I am following voluntary restraint Woodcock are present but I have imposed voluntary restraint on the shooting of them	50	70
I do not have access to woodcock shooting Woodcock are not present and I do not provide woodcock shooting	22	4

## 4 DISCUSSION

### 4.1 Overall summary

Using a data triangulation approach we could combine qualitative and quantitative datasets to assess changes in attitudes and approaches to shooting woodcock over time. When assessed collectively, these five data sources indicate that there has been a change in the behaviour of hunters in the UK harvesting woodcock over time. The decline in woodcock being harvested reported in Aebischer (2019) between 2004 and 2016 appears to continue, with the current estimate of 62,000 being almost one‐third of that 20 years ago. Woodcock shooting appears to be a relatively niche part of UK gamebird shooting with less than 30% of shoots offering them as quarry (with <3% of shoots offering them as a main quarry), perhaps 20%–30% of hunters shooting any woodcock in a season, and ~4% of hunters seeking woodcock as a main quarry species. These figures may be driven by self‐restraint practised by hunters or shoot providers with 36%–70% choosing not to shoot/offer shooting and 21% of hunters choosing to stop shooting woodcock despite having initially done so. There has also been a marked change in when woodcock are being shot, with around a 10‐fold decrease from the early to mid‐twentieth century when >20% of woodcock were shot early in the season, before 1st December, compared to the start of the twenty‐first century when around 3% of woodcock were shot before 1st December. This change coincides with, and precedes, advice from the GWCT issued from 2015 to 2018 regarding voluntary restraint in shooting woodcock.

#### 4.1.1 What is the current scale and extent of woodcock shooting in the UK?

Woodcock shooting currently comprises a small portion of gamebird hunting in the UK, with relatively few birds harvested, few hunters targeting them on generic shoot days and few species‐specific shoots targeting them across the UK. The data from the advertising game shoots on GunsOnPegs, the self‐report data from providers in the Value of Shooting survey and individual hunter gamecard records indicate that just over a quarter (26%–29%) of shoots either advertise or offer woodcock as quarry or record harvesting them. Madden (2021) estimated that there were ~3500–9000 shoots in the UK, suggesting that woodcock are harvested at 1000–2500 locations in the UK. Even on these shoots, woodcock typically appear to be an infrequent quarry, with the gamecard data revealing that one or more woodcock are harvested on a shoot on 18% of days when hunting occurred there. Only 11% of hunters in the 2016 GunsOnPegs survey reported having harvested woodcock as one of their three main quarry species in the preceding year, and 33% of hunters in the 2018 GunsOnPegs survey reported harvesting any woodcock in the preceding year. The total number of woodcock being harvested is relatively small and appears to be falling. In 2016, the harvest was estimated to be 140,000 (95% CI 120,000–170,000; Aebischer, 2019). From our gamecards, woodcock comprise <1% of the total bag, and if this figure is applied to the most recent estimate of released gamebirds (Madden, 2021) with a harvest efficiency of 35% (Robertson et al., 2017), then around 107,000 (95% CI 72,000–142,000) woodcock are harvested annually. The Value of Shooting report, based on data from 2021 to 2022, indicates that 62,000 (51,000–73,000) woodcock were harvested annually.

Despite these generally low figures, there is a strong skew in the distribution of woodcock hunting, with a small number of hunters and shoots specialising in them as a quarry species. From our gamecard data, <1% of shoot days were what we considered to be ‘woodcock specific’. This corresponds well to the figure of <3% of lowland shoots advertising on GunsOnPegs specialising in woodcock and 4% of hunters responding to the 2016 GunsOnPegs survey preferentially targeting woodcock as quarry. Therefore, the actions of a small number of individuals at a few locations may have a disproportionate effect on the national harvest and compliance patterns, and it is these individuals and operations where behaviour change could be most influential.

#### 4.1.2 Is there evidence of current self‐regulation of woodcock harvest in the UK?

Both our quantitative and qualitative data sources suggest that there is widespread knowledge of, and compliance with, woodcock shooting guidance among hunters and providers of shooting. Survey responses reveal that over 90% of hunters and shoot providers (that responded to the question) reported choosing to shoot woodcock only after 1st December or not at all (Table 4). This might indicate restraint, likely motivated by education and ecological understanding, because of those that shoot woodcock, 84% reported doing so ‘only when it is clear there is good understanding of the local population’ (GunsOnPegs shoot census data). It would be worth further exploration of where hunters acquired guidance and how effective different sources might be, including whether exposure and uptake differ according to the form and scale of hunting that an individual practices. This self‐report data was supported by the harvest data, with the percentage of woodcock harvested before 1st December being <3% since 2008 (gamecard data) or 13% since 2017 (wing record data), and with 12% of shoots where any woodcock are harvested, harvesting them before 1st December (gamecard data).

However, this change in patterns of harvest might also be explained by changes in woodcock populations, arrival or movement in the UK rather than deliberate self‐restraint. It might be that historically, woodcock shot early in the season (e.g. before 1st December) were resident birds, and due to their recent population declines (Balmer et al., 2013) there are simply proportionately fewer resident birds available to shoot. Little is known about the timings of winter migration into the UK, but it is possible that due to global warming, these migrations start later so that woodcock arrive later in the UK, or disperse later to north and western regions where much woodcock shooting occurs, meaning that there are fewer migratory woodcock available to shoot (as well as fewer residents) early in the season, resulting in fewer being shot at this stage. A better understanding of the origins of the harvested woodcock at different times of the year, and a study of patterns of winter migration into the UK over time would help evaluate these alternative explanations.

#### 4.1.3 How has woodcock harvest behaviour changed over time, and has this change coincided with advice and education interventions?

There has been little change in the proportion of lowland driven shoots in the UK where woodcock are harvested or in their proportion of the total bag of gamebirds harvested (gamecard data) over the past century. This may change in coming years given that a third of hunters declare that they have now stopped shooting woodcock with 11% having stopped in the past 3 years (GunsOnPegs shoot census data). There are indications (see §1 above) that since 2004, the number of woodcock harvested in the UK may have fallen by up to two‐thirds. However, this fall may coincide with a general decrease in overall shooting effort and gamebirds harvested, partly driven in recent years by disruption from COVID‐19 and avian influenza. These crude indications of reduced hunter effort may mask more subtle patterns. For example, we do not know whether those hunters who have ceased shooting woodcock were those who shot few anyway, with the dedicated woodcock specialists continuing their harvests, such that any population‐level effect is likely to be small. Additionally, commercial woodcock specialist shoots may cater to international clients who were not covered by the surveys that we used. A better and more fine‐scale understanding of the motivations and actions of those who do or did shoot woodcock is required to predict the consequences of these apparent behaviour changes.

The change that would most strongly indicate behaviour change is in the proportion of all woodcock harvested annually that were harvested before 1st December. The advice from the GWCT given in 2018 was that woodcock shooting should not begin until after the migrant birds have arrived, or after the 1st December (Brewin et al., 2022). When migrant woodcock arrive is likely to vary on a local level, depending on moon, weather and conditions in the wintering grounds (Powell, 2013), making assessment highly site‐specific. The vast majority of hunters that target woodcock report that they shoot them only when there is a clear understanding of the local population (GunsOnPegs shoot census data), suggesting that self‐restraint is influenced by some level of ecological understanding. A simpler rule‐of‐thumb that does not take account of local conditions suggested by GWCT is to not harvest woodcock until after 1st December. Therefore, birds harvested prior to December do not necessarily indicate non‐compliance with the voluntary advice if the hunter accounted for local conditions, and most hunters report that they do account for local conservation status, if they shoot woodcock at all (GunsOnPegs shoot census data). If combined with more detailed bag data, further analysis of annual trends, such as the significant increase in birds harvested in November 2018 (Table 2), may shed light on the impact of weather on the timing of migration and hence harvest.

### 4.2 General discussion

Successful, long‐term conservation of species and habitats, is to an extent, driven by human behaviour rather than by policy and legislation. In the case of wildlife management and hunting, policy‐makers must take into account the traditional and social aspects of actions, as well as motivations surrounding wildlife management and how this may influence behaviour change and compliance (Wallen & Daut, 2018). The value of behaviour change has been highlighted across a range of campaigns, from reducing single‐use plastics (Mathew et al., 2023), improving water conservation (Liyanage & Vishwanathan, 2020) and reduction in the illegal trade of wildlife (Wallen & Daut, 2018).

Policing and enforcement of legislation restricting woodcock shooting would be difficult, with the activity commonly occurring on remote private land with restricted public access and visibility, often involving only small groups of individuals and with little throughput of harvested birds to game dealers. Therefore, change might be better achieved by messaging and education from individuals and organisations within the hunting community. This makes it imperative that woodcock hunters take ownership of this issue and exhibit and enact the behavioural change required. Autonomy, or ownership of the responsibility to change, has been highlighted across a number of studies as a major driver of success (Black, 2001; Cromie et al., 2014; Mathew et al., 2023). However, polarisation and stakeholder conflict can be destructive to the process of change, resulting in the erection of sociological and political barriers, polarised loyalties and mistrust between interested parties (Cromie et al., 2014). A divisive, enforcement‐led approach to woodcock conservation by non‐hunting organisations risks being detrimental to long‐term efforts to improve compliance with best‐practice hunting behaviour, whether this be restraint in the numbers harvested or the period when harvest occurs, and thus to secure conservation goals.

If voluntary regulations align with management objectives of regulators, they could be considered as an alternative to legal regulation, rather than a stepping stone towards it. Not only are sector‐led changes lower‐cost than formal (policy‐led) regulation, but stakeholder engagement with management processes can lead to greater trust and better relationships with regulators, enabling collaborative working towards management targets (Cooke et al., 2013). Given the practical difficulties of enforcing legislation (see above), self‐regulation may provide the only feasible mechanism to change hunter behaviour. As with formal fishing regulations, a ‘one‐size‐fits‐all’ approach to wild bird harvest is likely to be inappropriate and can become complex to manage if transferred to a local or regional context, leading to poor compliance. With self‐enforced informal management, as appears to be evident in the case of woodcock shooting in the UK, the regulation of certain practices may be simplified to better fit local stakeholders and ecological contexts, leading to greater compliance.

## AUTHOR CONTRIBUTIONS

**Cat M. McNicol:** Conceptualization (equal); data curation (equal); writing – original draft (equal); writing – review and editing (equal). **Matt B. Ellis:** Conceptualization (equal); data curation (equal); writing – original draft (equal); writing – review and editing (equal). **Joah R. Madden:** Conceptualization (equal); data curation (equal); formal analysis (lead); writing – original draft (equal); writing – review and editing (equal).

## CONFLICT OF INTEREST STATEMENT

CMM and MBE are both employees of BASC, a membership organisation that promotes shooting sports in the UK. Their time on this project was part of their salaried work for BASC. JRM has conducted contract research work for BASC, with payment accruing to his University of Exeter research account, but not his personal account. JRM received no funding from BASC to conduct this piece of work. JRM's time on this project was part of his designated research time supported by the University of Exeter.

## Supporting information


Data S1.

## Data Availability

The gamecard data (fully anonymised) is available on Dryad (Madden et al., Forthcoming). A case study on the voluntary restraint of woodcock hunting in the UK [Dataset]. Dryad. doi.org/10.5061/dryad.8gtht76x7. All other survey data is presented within the manuscript.
